# Supplementary material for: Complexome profiling of the Chlamydomonas psb28 mutant reveals TEF5 as an early PSII assembly factor
Source: Plant Cell. 2025 Mar 17;37(6):koaf055. doi: 10.1093/plcell/koaf055 (PMC12164590; doi:10.1093/plcell/koaf055)
Supplement: koaf055_Supplementary_Data [file koaf055_supplementary_data.zip › psb28_interactive.zip!/plots/intensity/qualityPlots/peptides/CGLD32_Cre12.g555400.t1.2.html]

### Cre12.g555400.t1.2

Trivial name: CGLD32  
SeqCoverage [median]: 9.30  
Mass: 20.70 kDa  
  
 

| Sequence | MissedCleavage | Mass [Da] | wtIntensity | muIntensity | Score |
| --- | --- | --- | --- | --- | --- |
| IYEAAQAEK | 0 | 1021.51 | 1868.00 | 9472.59 | 85.68 |
| YAEATNNPR | 0 | 1034.48 | 1466.21 | 5052.00 | 107.66 |
|  |  |  | 3334.2 | 14524.6 |  |
